# Assessment of soil nitrogen and related enzymes as influenced by the incorporation time of field pea cultivated as a catch crop in *Alfisol*

**DOI:** 10.1007/s10661-014-4014-0

**Published:** 2014-09-06

**Authors:** Anna Piotrowska-Długosz, Edward Wilczewski

**Affiliations:** 1 Department of Soil Science and Soil Protection, Division of Biochemistry, Faculty of Agriculture and Biotechnology, University of Technology and Life Sciences, Bernardyńska 6 St., 85-029 Bydgoszcz, Poland; 2 Department of Agrotechnology, Faculty of Agriculture and Biotechnology, University of Technology and Life Sciences, Kordecki 20 St., 85-225 Bydgoszcz, Poland

**Keywords:** Catch crops, Autumn incorporation, Spring application, N-cycle enzymatic activity, Mineral N, Chemical properties

## Abstract

The effect of the time of catch crop (field pea) incorporation [catch crop incorporated in the autumn (A) or in the spring (B) versus plots without a catch crop (C)] on the soil enzymes related to N transformation (urease – UR, protease – PRO, nitrate reductase – NR, arginine ammonification rate – AAR), the total N and mineral N as well as microbial biomass N (MBN) contents were investigated in a 3-year experiment. The catch crop was sown at the beginning of August and plowed in the autumn in 2008, 2009 and 2010 or left as mulch during the winter. Soil samples for microbial activity were taken from spring barley plots that were grown in 2009, 2010 and 2011 before sowing (March), during the tillering phase (May), shooting (June) and after the harvesting of spring barley (August). The use of catch crop significantly increased the soil mineral and MBN contents as well as the activities of PRO and NR as compared to the control soil. The spring incorporation of the field pea significantly increased the MBN content in contrast to the autumn application, while the activity of N-cycle enzymes were clearly unaffected (UR and AAR) regardless of the time of the incorporation of field pea or else the results were inconsistent (PRO and NR). When the catch crop was incorporated in the spring, a significantly higher content of mineral N as compared to autumn incorporation was noted on only two of the four sampling dates. The enzymatic activity (PRO and AAR) was about 1.3-2.8 times higher in May and June as compared with March and August. Both spring or autumn incorporation of catch crop can be a useful management practice to increase the soil mineral N content and enhance the soil biological activity.

## Introduction

Nitrogen fertilization has played a major role in the global food production over the past sixty years, and about fifty percent of total N comes from fertilizer supply (Zhaohui et al. 2012). The effectiveness of nitrogen utilization from fertilizers is, however, very low and does not exceed 10–50 percent for crops grown in fields (Ribaudo et al. 2011). The reasons for this low efficiency are the loss of N through leaching, runoff, ammonia volatilization or denitrification, which results in the pollution of the groundwater and atmosphere (Zhaohui et al. 2012; Zarabi and Jalali 2012). The main cause of the loss of N in drainage is through the leaching of nitrate. Most nitrate leaching occurs during the autumn/winter drainage period, though nitrate can be lost at any time if there is sufficient rain to completely wet the soil (Shepherd et al. 2003). The loss of N occurs when a crop is supplied with more N than it needs or as a consequence of a lack of synchrony between the N supply and crop N requirement, e.g., if plowed grass residues are mineralized after the crop has matured (Briggs et al. 2005). One of the strategies that is used to prevent N losses in intensive agricultural systems is the cultivation of catch crops and using them as green manure (Tripolskaya et al. 2004). Although catch crops are grown for many reasons, most of the interest is focused on their effect on nitrogen. The incorporation of a biomass of catch crops increases the immobilization of soil nitrogen (N), prevents leaching losses of N into the environment and improves the N supply for succeeding crops (Thorup-Kristensen et al. 2003; Thomsen 2005). Leguminous catch crops, which provide a substantial amount of biologically fixed N to the primary crop are especially valuable. Additionally, the decomposition of their organic matter is easy due to their low carbon to nitrogen (C:N) ratio (Fageria et al. 2005).

One of the possible options to improve the N effect of catch crops is the choice of the incorporation time. The N that is retained in catch crops can be made available for the succeeding spring cereals by timing the soil incorporation of the catch crop relative to the sowing of a cereal in order to allow decomposition of the catch crop residues (Doltra and Olesen 2013). Catch crops, incorporated either in the autumn or the spring, have previously been compared in order to find the optimal incorporation time (Hansen et al. 1997; Thomsen 2005). In some other experiments two or three incorporation dates in the spring (Garwood et al. 1999) or in the autumn (Torstensson 1998; Wallgren and Lindén 1994) have been considered.

The catch crop is effective in reducing nitrate leaching most often when incorporated in the spring (Hansen et al. 1997). As stated by Thorup-Kristensen and Dresbøll (2010), spring incorporation, compared to the autumn one, will allow more time for the catch crop to uptake N and will reduce the risk that the N that is mineralized from the catch crop will be lost through leaching before it can be taken up by the succeeding crop. According to these authors, the greatest mineralization effect of a catch crop was noted after the early spring incorporation. However, if catch crops are allowed to grow in the winter, the yield of the following crop may be reduced, e.g. due to the phytotoxicity of some catch crops (Garwood et al. 1999). Furthermore, the reduction in yield may be the result of the depletion of available N in the soil by catch crops compared with no catch crop, especially when the spring time of incorporation is delayed (Thorup-Kristensen et al. 2003). The incorporation of catch crops in the autumn followed by a mild winter may also reduce their effect of N uptake and increase the leaching of the N that is mineralized from the catch crop (Thorup-Kristensen and Dresbøll 2010).

Some studies have been devoted to the important role of catch crops in increasing the productivity of subsequent crops by improving the soil’s physical (Chirinda et al. 2010), chemical (Arlauskiené and Maikšténiené 2010) and biological properties (Navas et al. 2011; Piotrowska and Wilczewski 2012). The incorporation of catch crops into the soil stimulates the growth and activity of the microbial communities (Stark et al. 2007) that are the main source of enzymes in soil. Soil enzymes are known to be involved in nutrient cycling, and as such, their activities can be used as potential indicators of the nutrient cycling processes (Janušauskaité et al. 2013). The enzyme ureaze catalyses the hydrolysis of urea to CO_2_ and NH_3_ with the concomitant rise in soil pH. This in turn, results in a rapid N loss to the atmosphere through NH_3_ volatilization. With respect to this, urease activity plays an important role in the regulation of N supply to plant after urea fertilization (Fazekašová 2012). Nitrate reductase activity (NR) is an important enzyme in the process of denitrification, which catalyzes the reduction of NO_2_^−^ to N_2_O under anaerobic conditions. Nitrate reductase is an adaptive enzyme and is synthesized only in the presence of NO_3_^−^ ions and that why its activity is commonly used as an indicator of the ability of plants to utilize NO_3_^−^ from the soil (Barford and Lajtha 1992). The availability of organic N for plants depends on the rate of its mineralization. Protein that is added to soil is readily decomposed by proteases and peptidases into smaller, membrane-permeable peptides and amino acids. The latter are further metabolized with the release of NH_4_^+^ (Ladd and Jackson 1982). Proteolysis is an important process in many ecosystems with regard to N-cycling because it is considered to be a rate-limiting step during N mineralization in soils due to the much slower primary phase of protease activities during N mineralization compared with amino acids mineralization (Jan et al. 2009). Alef and Kleiner (1986) have suggested a method of determination of potential microbial activity by using arginine ammonification rate. The arginine is mineralized to ammonium and the content of ammonium is extracted from soil and measured. The arginine ammonification rate was proportional to the amount of soil microbial biomass and was proposed to be used as a method to estimate soil microbial biomass activity (Alef and Kleiner 1986).

The soil microbial biomass, the living part of the soil organic matter, functions as a transient nutrient sink and is responsible for the decomposition and transformation of organic materials, which are mostly derived from aboveground and below-ground plant residues (Ananyeva et al. 1999).

With that in mind we assumed that (1) the soil nitrogen, especially N mineral forms, and the activities of N-cycle enzymes would be increased by field pea (FP) (*Pisum sativum* L.) cultivated as catch crop as compared to the control, (2) the time of the incorporation of the catch crop would differentiate the mineral N content and the activity of N-cycle enzymes and (3) significant relationships between the enzymes being studied and chemical properties could be expected. In order to test our hypotheses, we assessed the content of total nitrogen (N_TOT_), the microbial biomass N (MBN), the mineral forms of N (N-NO_3_^−^, N-NH_4_^+^) and the activities of four N-related soil enzymes that are affected by the field pea incorporation time versus the control soil.

## Material and methods

### Site description and experimental design

The effect of the incorporation time of the catch crop on the soil N forms and the activities of N-related enzymes were studied for three years (2009–2011) in a field, one-factor experiment carried out in a randomized block design with four replications at the Experimental Station in Mochełek (17^o^ 51’ E; 53° 13’ N) near Bydgoszcz (Midwestern Poland). The time of catch crop incorporation was the experimental factor. The catch crop green mass was incorporated in the autumn (A) and in the spring (B), while the control soil was tilled without the catch crop (C). Every year the experiment was performed in a different parts of the experimental field. They were situated close to each other to minimize soil spatial variability. Soil samples were collected twice per year in 2009, 2010 and 2011 in order to determine its chemical properties. Samples were taken in the spring, always before the sowing of spring barley (between 23^rd^ and 30^th^ of March) and in August, immediately after the harvest (between 10^th^ and 15^th^ of August). Moreover, four sampling dates were established in order to assess the seasonal variation in the soil mineral N content and the activities of N-related enzymes under spring barley: before sowing (March), during the tillering phase (May), during the shooting phase (June) and after the harvesting of spring barley (August). Soil samples were taken from a depth of 0–30 cm. Ten samples were collected randomly from each plot and bulked in order to provide one representative sample per plot.

Winter wheat (*Triticum aestivum* L.) was the crop before the field pea cultivated as a catch crop. The field pea was sown between 5^th^ and 9^th^ of August after the harvesting of winter wheat. Catch crops were harvested between 15^th^ October and 3^rd^ November (in 2008–2010) using a self-propelled mower. Then, the above-ground biomass was weighed and scattered on the soil surface on each plot. The yield of post-harvest residues was measured based on samples that were taken from soil monoliths 25x25x25 cm. Four monoliths were taken from each plot. The soil taken from the monoliths was sieved and rinsed in water. The remains of weeds and straw that had not decomposed were removed manually. Then the post-harvest residues were weighed, dried at 50 °C and reweighed. The soil in plots A and C was then plowed (at a depth of 27 cm). The field pea in plot B was left to grow in the winter. Frosts that occurred in December and January resulted in the aboveground biomass freezing. When this happened, the biomass was left on the surface of the soil through the winter. It was cut up and mixed with soil (at a depth of 10–12 cm) using a disc harrow the following spring. Spring barley (*Hordeum vulgare* L.) was sown between 2^nd^ and 8^th^ of April of the following years (2009–2011).

Phosphorus (P) as Ca(H_2_PO_4_)_2_ and potassium (K) as KCl were applied in the spring at doses of 26.2 kg ha^−1^ and 66.4 kg ha^−1^, respectively. Nitrogen fertilization as NH_4_NO_3_ (90 kg ha^−1^ N) was applied in two doses: 45 kg ha^−1^ was applied before the sowing of spring barley (together with K and P fertilization) and 45 kg ha^−1^ was applied during the shooting of spring barley. The same management procedures were repeated each year during the entire study period (2009–2011).

According to the USDA Soil Taxonomy (Soil Survey Staff 2010), the soil that was studied was a typical *Alfisol* formed of a sandy loam (clay 6 %, sand 79 %, silt 15 %). The selected chemical properties of the soil that were determined before the experiment are presented in Table 1.

**Table 1:** Basic soil characteristics before the sowing of catch crop (0–30 cm)

Properties	Years		
	2008	2009	2010
C_ORG_ (g kg^−1^)	7.6	6.8	11.9
N_TOT_ (g kg^−1^)	0.63	0.60	1.11
pH_KCl_	5.65	5.70	6.86
Soil moisture (%)	6.58	7.06	7.14

The field experiment was carried out in a region with a temperate, changeable climate where the marine air from the North Atlantic and continental air from the east converge, thus causing frequent day-to-day and year-to-year variability in the weather patterns. Detailed data about the weather conditions during the sampling periods were obtained from a local weather station that is located at the Experimental Station at Mochełek and are presented in Table 2. The average annual temperature and rainfall during the period of the investigation (2009–2011) were about 12.3 °C and 432 mm.

**Table 2:** Mean air temperature and sum of precipitation at the experiment site

Months	2008	2009	2010	2011	2008	2009	2010	2011
	Precipitation (mm)				Temperature (°C)			
January	48.2	14.2	22.0	33.0	0.5	−3.3	−7.8	−0.6
February	15.9	19.4	20.1	14.5	2.8	−0.9	−2.7	−4.7
March	61.2	43.7	28.6	11.7	3.0	2.4	2.4	2.2
April	38.7	0.4	33.8	13.5	7.6	9.8	7.8	10.5
May	11.5	85.3	92.6	38.4	13.2	12.3	11.5	13.5
June	15.5	57.4	18.1	100.8	17.6	14.5	16.7	17.7
July	58.7	118.0	107.4	132.5	19.2	18.6	21.6	17.5
August	95.5	17.6	150.7	67.7	17.8	18.2	18.4	17.7
September	20.2	34.4	74.7	37.0	12.4	13.7	12.2	14.3
October	19.4	40.4	2.3	13.2	8.4	6.3	5.5	8.4
November	80.0	66.2	115.0	9.0	4.3	5.2	4.1	2.7
December	24.8	35.4	39.9	46.2	0.2	−1.1	−6.7	2.7

### Laboratory analysis

The chemical properties of the soil were assessed in triplicate according to standard methods. Total nitrogen (N_TOT_) in the soil was determined using the Kjeldahl method (Bremner and Mulvaney 1982). Soil organic carbon (C_ORG_) content was determined using the dichromate oxidation procedure, while soil pH (1 M KCl) was measured using the potentiometric method in 1:2.5 soil:solution suspensions. Soil moisture was analyzed using drying-weighing method. The content of N-NO_3_^−^ and N-NH_4_^+^ were extracted from field-moist soil with KCl and K_2_SO_4_, respectively. The nitrate nitrogen content was determined using the phenoldisulphonic acid method and the ammonium nitrogen concentration was assayed using the indophenol blue method (Bashour and Sayegh 2007). The Kjeldahl method was used to determine the total N of the aboveground biomass and post-harvest residue of catch crops after the mineralization of the shredded plant material by digestion in concentrated H_2_SO_4_ and H_2_O_2_.

All assays of enzyme activities were performed on fresh, moist, sieved (<2 mm) soils. Triplicates were performed for each activity assay. All enzyme activity values were calculated based on the oven-dry (105 °C) weight of soil.

Casein-protease (PRO) activity was assayed using the Ladd and Butler method (1972). Briefly, 2.5 ml of Tris-buffer (0.2 M, pH 8.0) and a 2 % Na-caseinate solution were added to 1 g of moist soil. The samples were incubated at 40 °C for 2 h. After the incubation, the remaining casein was precipitated with 5 ml of cold 10 % trichloroacetic acid. The suspension was then filtrated and 0.75 ml Na_2_CO_3_ (1.4 M) and 0.25 ml three-fold diluted Folin-Ciocalteu reagent were added to 0.5 ml of the filtrate and mixed. The tyrosine concentration was measured photometrically at 680 nm and was expressed as mg TYR kg^−1^ h^−1^.

The arginine ammonification rate (AAR) was measured using the Kandeler method (1995). After the addition of an aqueous L-arginine solution (11.5 M), soil samples were incubated for 3 h at 37 °C. After the incubation, the ammonium released by AAR was extracted with 2 M KCl by shaking for 30 minutes and the soil suspension was filtrated. For photometric analysis at 630 nm, the filtrate was mixed with a sodium phenolate solution (0.12 M), a sodium nitropruside solution and sodium hypochlorite and allowed to stand for 30 minutes at room temperature for color development. One unit of arginine ammonification rate was defined as the number of mg of product released by 1 kg of dried soil at 37 °C per 1 hour (mg N-NH_4_^+^ kg^−1^ h^−1^).

Soil urease activity was assayed as described by Kandeler and Gerber (1988). Briefly, 1 g of moist soil was incubated with 4 ml of a borate buffer (pH 10.0) and 0.5 ml of a urea solution in reaction flasks for 2 h at 37 °C. After the incubation, 6 ml of 1 M KCl was added to all of the flasks for 30 minutes. To assess the ammonium content, the filtrate was mixed with water, Na salicylate/NaOH and sodium dichloroisocyanide and allowed to stand at room temperature for 30 minutes prior to measuring the optical density at 690 nm. The activity of UR was expressed as the AAR.

Nitrate reductase activity (NR) was determined according to Kandeler (1995). Field moist soil samples were incubated for 24 hours at 25 °C with a 0.9 mM 2,4-DNP (dinitrophenol) solution, a substrate (25 mM KNO_3_) and distilled water. After incubation 10 ml of a 4 M KCl solution was added to both the samples and controls; the contents of test tubes were mixed briefly and filtered immediately. For spectrophotometric analysis, 5 ml of filtrates, 3 ml of an ammonium chloride buffer (0.19 M, pH 8.5) and 2 ml of a color reagent were added to the test tubes and mixed. The extinction was measured at 520 nm against the reagent blank. Values of NR activity were expressed as mg N-NO_2_^−^ kg^−1^ 24 h^−1^.

The microbial biomass N was determined using the chloroform fumigation-extraction method (Brookes et al. 1985). Moist soil samples (25 g) were fumigated with ethanol-free CHCl_3_ at 25 °C for 24 h. The fumigated and unfumigated soils were extracted with 0.5 M of K_2_SO_4_ at a ratio 5:1. Then the soil suspension was centrifuged at 200 rev min^−1^ for 30 minutes and filtrated. Determination of the total N from both the fumigated and unfumigated soils was done according to Bremner and Mulvaney (1982). To account for an incomplete recovery of microbial N, the microbial biomass was calculated by dividing the difference between the fumigated and unfumigated samples by a correction factor of 0.54 (=k_EN_) (Joergensen and Mueller 1996).

### Statistical analysis

A two-way analysis of variance (ANOVA) was performed to examine the effect of catch crop management (spring incorporation, autumn incorporation, control) and the dates of sampling (seasonal variation) on the properties studied. In the case of significant *F*-tests, differences between the group means were assessed using the Tukey test (*P* <  0.05). Simple and multiple regressions were done to show the relationship among the properties studied. All of the statistical analyses were conducted using Statistica 8.1 for Windows software.

## Results

### Catch crop biomass and nitrogen content

**Fig. 1:**
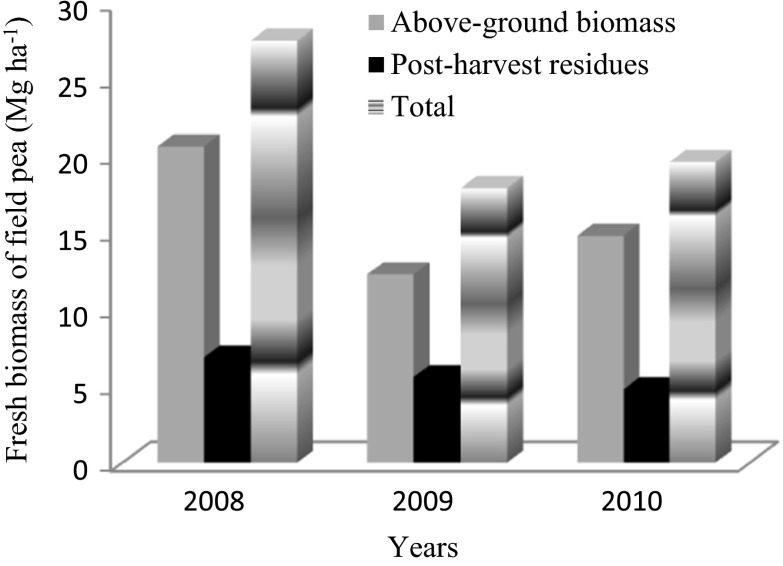
Fresh biomass of field pea (Mg ha^−1^) during the experiment (2008–2010)

Fresh biomass of field pea grown as a catch crop was presented on Fig. 1. The highest content of both above-ground biomass and post-harvest residues of field pea was noted in 2008 followed by 2009 and 2010. A higher concentration (% of dry weight) of total N in the above-ground biomass of catch crop was noted in 2010 (3.50 %) than in 2008 and 2009 (average of 2.94 %), while in the harvested residues, a lower total N content was noted in 2008 (1.84 %) compared to 2009 and 2010, where an average content of 2.54 % was assayed (Table 3). The total content of nitrogen taken up by the catch crop ranged from 66.4 kg ha^−1^ in 2009 to 112.6 kg ha^−1^ in 2008 (Table 3). The 74–82 % of the nitrogen that was taken up was accumulated in the green mass of the field pea and only 18–26 % of this element was accumulated in the post-harvest residues of this plant. This was due to both the differences in the mass of the individual parts of the plant (Fig. 1), as well as due to the higher concentrations of this element in the green mass in contrast to the post-harvest residue The highest values of soil properties (C_ORG_, N_TOT_, pH) in the soil before the catch crop was sown in 2010 (Table 1) corresponded with the highest percentage of N in the aboveground biomass of the catch crop in that year and was higher than in the 2009 N uptake (Table 3).

**Table 3:** The content and the uptake of N by the catch crop

Kind of biomass	The content (%)			The uptake (kg ha^−1^)		
	2008	2009	2010	2008	2009	2010
Above-ground biomass	2.98	2.89	3.50	92.4	49.1	70.0
Post-harvested residues	1.84	2.47	2.60	20.2	17.3	20.8
Mean	2.41	2.68	3.05			
Total				112.6	66.4	90.8

### Mineral N and other chemical properties of soil

Both the ammonium-N (N-NH_4_^+^) and nitrate-N (N-NO_3_^−^) content was significantly affected by the catch crop management and sampling season in individual years of the study (Tables 4, 5 and 6), but there were no consistent trends in both mineral N forms that were dependent on factors examined. Therefore, the results of N-NH_4_^+^ and N-NO_3_^−^ are additionally shown as the average for the entire experimental period (2009–2011), in order to make the data clearer and to easily find out whether any general tendency in their changes occurred during the experimental period.

**Table 4:** *P* values deriving from two-way ANOVA applied to studied chemical and biological variables, using catch crop management and sampling season as factors (2009–2011). NS: not significant

Variables	*P*_catch crop management (M)_	*P*_season (S)_	*P*_M x S interaction_
C_ORG_ (g kg^−1^)	NS	0.034	NS
N_TOT_ (g kg^−1^)	NS	NS	NS
pH_KCl_	NS	NS	NS
MBN (mg kg^−1^)	0.007	NS	NS
N-NH_4_^+^ (mg kg^−1^)	0.012	0.002	NS
N-NO_3_^−^ (mg kg^−1^)	0.001	0.006	0.026
UR (mg N-NH_4_^+^ kg^−1^ h^−1^)	0.034	NS	NS
AAR (mg N-NH_4_^+^ kg^−1^ h^−1^)	0.031	0.019	NS
PRO (mg TYR kg^−1^ h^−1^)	0.011	0.005	NS
NR (mg N-NO_2_^−^ kg^−1^ 24 h^−1^)	0.019	0.028	NS

**Table 5:** Ammonium-N content in soil (mg kg^−1^) as dependent on the time of catch crop incorporation and sampling season

Catch crop	^§^March	May	June	August
2009				
A^&^	4.93ab^B^#^	3.87bB	9.05aA	
B	5.44aB	5.94aB	8.05aA	
C	3.87bA	2.67bA	4.37bA	
Mean	4.75B	4.16B	7.16A	
2010				
A	3.06aB	7.57bA	7.39aA	4.20aB
B	3.77aB	10.6aA	8.41aA	5.20aB
C	3.56aB	8.10bA	5.29bB	3.93aB
Mean	3.46B	8.76A	7.03A	4.44B
2011				
A	4.33aA	2.61aB	3.26aAB	2.48bB
B	3.51aAB	3.72aA	2.32abB	4.15aA
C	2.45bA	2.29bA	1.50bA	2.70bA
Mean	3.43A	2.87AB	2.36B	3.11A
2009–2011				
A	4.11aB	4.68bAB	6.57aA	3.34^§§^bB
B	4.24aB	6.75aA	6.26aA	4.68aB
C	3.29bB	4.35bA	3.72bAB	3.32bB
Mean	3.88B	5.26A	5.52A	3.78B

^&^ A – catch crop incorporated in autumn, B – catch crop incorporated in spring, C – control (without a catch crop); ^§^ sampling dates; ^§§^ mean of 2010 and 2011.

Values followed by the same small letter within each column are not significantly different at *P* <  0.05. Values followed by the same capital letter within a line are not significantly different at *P* <  0.05.

^ Different small letters indicate comparison between catch crops treatments (within the same sampling date).

^#^ Different capital letters indicate a comparison among sampling dates within the same catch crop treatments.

**Table 6:** Nitrate-N content (mg kg^−1^) as dependent on the time of catch crop incorporation and sampling season

Catch crop	^§^March	May	June	August
2009				
A^&^	2.71b^B^#^	4.83aA	4.45aAB	
B	6.28aA	1.82bB	3.33abB	
C	1.92bA	1.66bA	2.54bA	
Mean	3.64A	2.77B	3.44A	
2010				
A	5.21bB	13.0bA	5.03bB	3.99aB
B	7.68aB	16.4aA	7.63aB	3.77aC
C	3.67bB	13.6bA	3.56bB	3.72aB
Mean	5.52B	14.3A	5.41B	3.83B
2011				
A	15.2aB	24.0bA	5.71aC	6.39aC
B	18.7aB	36.3aA	6.07aC	8.65aC
C	9.16bB	17.4cA	4.73aB	6.05aB
Mean	14.4B	25.9A	5.50C	7.03C
2009–2011				
A	7.71bB	13.9bA	5.06aB	5.19^§§^abB
B	10.9aB	18.2aA	5.68aC	6.21aC
C	4.92cB	10.9cA	3,61bB	4.89bB
Mean	7.84B	14.3A	4.78C	5.43C

^&^A – catch crop incorporated in autumn, B – catch crop incorporated in spring, C – control (without a catch crop); ^§^ sampling dates; ^§§^ mean of 2010 and 2011.

Values followed by the same small letter within each column are not significantly different at *P* <  0.05. Values followed by the same capital letter within a line are not significantly different at *P* <  0.05.

^ Different small letters indicate comparison between catch crops treatments (within the same sampling date).

^#^ Different capital letters indicate a comparison among sampling dates within the same catch crop treatments.

The N-NH_4_^+^ content was within a broad range of 1.50-10.6 mg kg^−1^ with a mean of 4.61 mg kg^−1^ (Table 5). Generally, the highest N-NH_4_^+^ concentration (average for 2009–2011) was assessed during the tillering and shooting of spring barley (May and June), while a lower content was noted before its sowing (March) and after harvesting (August). In 2009 and 2011 there was no significant seasonal changes in the N-NH_4_^+^ content in the control soil. The concentration of N-NH_4_^+^ in soil in March and June was significantly higher than in the control in both catch crop treatments. During the tillering of spring barley (May) and after its harvesting, the content of ammonium-N in soil was higher in the soil that had been treated with mulch than in the control and in the treatment where catch crop was plowed in the autumn.

Nitrate-N ranged broadly from 1.66 to 36.3 mg kg^−1^ with a mean of 8.09 mg kg^−1^. In contrast to ammonium-N, the nitrate-N was highest in the last year of the experiment followed by 2010 and 2009 (Table 6). When the entire period of the experiment was considered together (2009–2011), the highest content of N-NO_3_^−^ (14.3 mg kg^−1^) was shown during the tillering of spring barley (May), while the lowest was observed during the shooting (June) and after the harvesting of spring barley (August) (average 5.11 mg kg^−1^). In May and March (2009–2011) the N-NO_3_^−^content was significantly higher when the catch crop was incorporated in the spring in contrast to the autumn incorporation and the control soil. In June (2009–2011) the influence of the time of the catch crop incorporation on the soil nitrate-N concentration was not significant, while in August the influence was inconsistent.

The time of the incorporation of the catch crop and the seasonal sampling did not significantly influence the N_TOT_ and pH_KCl_, while the C_ORG_ content was affected only in 2009 when it was higher in August in contrast to March (Table 4, Table 7). Both C_ORG_ and N_TOT_ were the highest in 2011 followed by 2010 and 2009. The soil reaction was acid in 2009 and 2010 and neutral in 2011.

**Table 7:** Basic soil characteristic during the experiment (2009–2011)

Properties	Sampling time	2009	2010	2011
C_ORG_ (g kg^−1^)	March	6.16^›^ (±0.52)b^	7.77(±0.43)	11.6(±0.52)
	August	7.77(±0.22)a	8.26(±0.40)	11.6(±0.68)
N_TOT_ (g kg^−1^)	March	0.55(±0.05)	0.63(±.0.04)	0.97(±0.04)
	August	0.58(±0.06)	0.64(±0.02)	1.10(±0.06)
pH_KCl_	March	5.47(±0.77)	4.75(±0.43)	6.66(±0.25)
	August	5.30(±0.65)	4.83(±0.40)	6.85(±0.15)

^›^mean (±standard deviation), ^ - values followed by different small letters within each column are significantly different at *P* <  0.05

### Microbial biomass N content and soil enzymatic activity

**Fig. 2:**
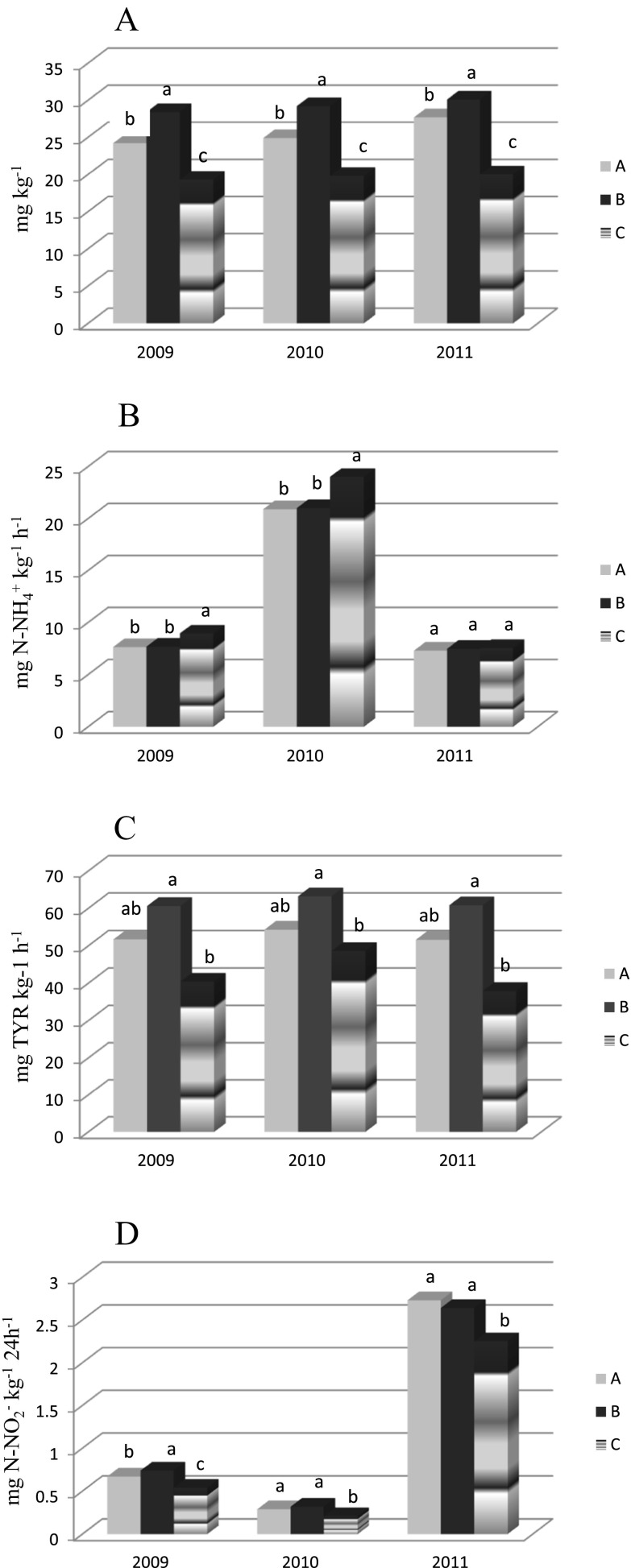
The content of MBN (**a**), urease activity (**b**), protease activity (**c**) and nitrate reductase activity (**d**) as dependent on the time of catch crop incorporation (average for four sampling dates). A – catch crop incorporated in autumn, B – catch crop incorporated in spring, C – control (without a catch crop). Values followed by the same small letter in the same year are not significantly different at *P* <  0.05

**Fig. 3:**
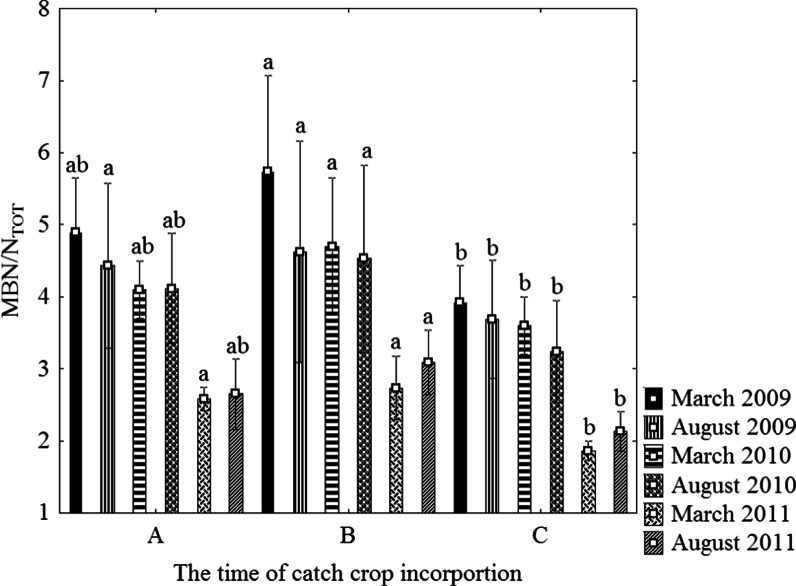
The MBN/N_TOT_ ratio as dependent on the time of catch crop incorporation and sampling date. Mean and standard deviation is given on each bar. Values followed by the same small letter in the same sampling date and different catch crop incorporation time (**a**, **b**, **c**) are not significantly different at *P* <  0.05

The MBN content differed significantly as a function of catch crop management while the influence of sampling dates and the interaction of both factors (M x S) was not significant (Table 4). Microbial biomass N (MBN) ranged between 19.3 and 30.1 mg kg^−1^ in the study (Fig. 2a). The highest MBN content was observed when the catch crop was incorporated in the spring (29.2 mg kg^−1^) followed by the indirect incorporation in the autumn (25.6 mg kg^−1^) and the control (19.7 mg kg^−1^). On average, approximately 3.6 % of total N was bound in the microbial biomass (Fig. 3). The biotic-fixed N differed significantly relative to catch crop management, while sampling dates (March and August) did not markedly influence the MBN content. A higher MBN/N_TOT_ ratio was noted when the catch crop was applied compared to the control soil.

The highest UR activity was found in 2010 (22.0 mg N-NH_4_^+^ kg^−1^ h^−1^), while in 2009 and 2011 the enzyme activity was lower (average 8 mg N-NH_4_^+^ kg^−1^ h^−1^) (Fig. 2b). In the two first years of the experiment, UR activity was significantly lower when the catch crop was applied as compared to the control, while there were no significant differences between the incorporation times of the catch crop (Fig. 2b). When the period investigated was considered together (2009–2011), the UR activity was the highest in May, lower in June, and the lowest in March and August (Table 8). Before the sowing of spring barley (March), the catch crop management did not influence the UR activity considerably, while on the succeeding sampling dates, the UR activity was significantly lower in the catch crop treatments than in the control (C).

**Table 8:** Urease activity (UR) (mg N-NH_4_^+^ kg^−1^ h^−1^) as dependent on the time of catch crop incorporation and sampling season during the experiment (2009–2011)

Catch crop	^§^March	May	June	August
A^&^	5.37a^C^#^	21.6 bA	13.2 bB	7.64 bC
B	5.49aC	21.2 bA	13.8 bB	7.70 bC
C	5.64aC	24.6 aA	15.2 aB	8.50 aC
Mean	5.50C	22.5A	14.1B	8.00C

^&^A – catch crop incorporated in autumn, B – catch crop incorporated in spring, C – control (without a catch crop); ^§^ sampling dates

Values followed by the same small letter within each column are not significantly different at *P* <  0.05. Values followed by the same capital letter within a line are not significantly different at *P* <  0.05.

^ Different small letters indicate comparison between catch crops treatments (within the same sampling date).

^#^ Different capital letters indicate a comparison among sampling dates within the same catch crop treatments.

**Fig. 4:**
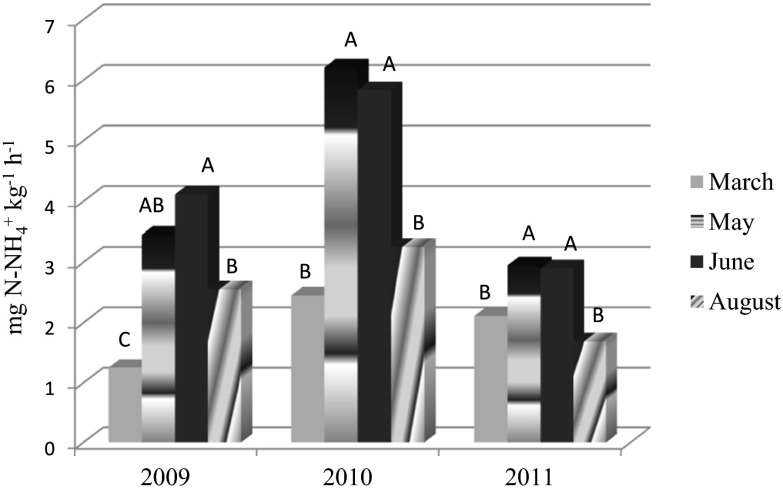
Arginine ammonification rate (AAR) as dependent on the sampling season during the experiment (average across treatments). Values followed by the same capital letter in the same year are not significantly different at *P* < 0.05

Similar to UR activity, the rate of the arginine ammonification rate (AAR) was the highest in 2010 (Fig. 4). The AAR was not significantly influenced by the catch crop management in individual years of the study. When the period investigated was examined together, the catch crop management influenced the AAR only in May and June as compared to the control, while there was no marked influence on this property in relation to the time of the catch crop incorporation (Table 9). The highest AAR was assessed during the tillering and shooting of spring barley (May, June), while lower activity was noted before its sowing and after harvesting (Table 9, Fig. 4).

**Table 9:** Arginine ammonification rate (AAR) (mg N-NH_4_^+^ kg^−1^ h^−1^) as dependent on the sampling season during the experiment (2009–2011)

Catch crop	^§^ March	May	June	August
A^&^	1.90a^B^#^	4.10bA	3.98bA	2.59aAB
B	1.85aB	4.11bA	4.07bA	2.51aAB
C	2.06aB	4.40aA	4.34aA	2.40aB
Mean	1.94B	4.20A	4.13A	2.50B

^&^A – catch crop incorporated in autumn, B – catch crop incorporated in spring, C – control (without a catch crop); ^§^ sampling dates

Values followed by the same small letter within each column are not significantly different at *P* <  0.05. Values followed by the same capital letter within a line are not significantly different at *P* <  0.05.

^ Different small letters indicate comparison between catch crops treatments (within the same sampling date).

^#^ Different capital letters indicate a comparison among sampling dates within the same catch crop treatments.

The casein hydrolyzing protease (PRO) activity ranged from 29.8 to 81.6 mg TYR kg^−1^ h^−1^ with a mean value of 51.9 mg TYR kg^−1^ h^−1^ (Table 10). Generally, the PRO activity was always higher in the catch crop treatments as opposed to the control (Table 10, Fig. 2c). The influence of the time of the catch crop incorporation on the PRO activity was inconsistent on most of sampling dates. The catch crop incorporated in the spring significantly increased the activity of PRO as compared with the autumn incorporation only in March 2009 and 2010 (Table 10). The enzyme activity was visibly higher in May and June followed by the activity in March and August in specific years of the study.

**Table 10:** Protease activity (PRO) (mg TYR kg^−1^ h^−1^) as dependent on the time of catch crop incorporation and sampling season

Catch crop	^§^March	May	June	August
2009				
A^&^	38.1b^B^#^	56.5abA	67.9abA	44.4abB
B	44.3aB	67.8aA	78.2aA	52.1aB
C	29.8bB	45.8bA	49.7bA	36.1bAB
Mean	37.4B	56.7A	65.3A	44.2B
2010				
A	40.8bB	60.7aA	74.3abA	41.5abB
B	52.3aB	69.9aAB	81.6aA	48.9aB
C	32.4cC	58.6bB	67.4bA	36.1bC
Mean	41.8B	63.1A	74.4A	42.2B
2011				
A	39.8abB	57.5abA	63.8aA	45.4abB
B	49.6aA	65.9aA	69.6aA	58.1aA
C	29.9bB	34.9bB	50.4bA	36.2bB
Mean	39.8B	52.8A	61.3A	46.6B
2009–2011				
A	39.6abB	58.2bA	68.7aA	43.8abB
B	48.7aB	67.9aA	76.5aA	53.0aB
C	30.7bB	46.4cAB	55.8bA	36.1bB
Mean	39.7B	57.5A	67.0A	43.3B

^&^A – catch crop incorporated in autumn, B – catch crop incorporated in spring, C – control (without a catch crop); ^§^ sampling dates

Values followed by the same small letter within each column are not significantly different at *P* <  0.05. Values followed by the same capital letter within a line are not significantly different at *P* <  0.05.

^ Different small letters indicate comparison between catch crops treatments (within the same sampling date).

^# ^Different capital letters indicate a comparison among sampling dates within the same catch crop treatments.

The NR activity across the period investigated ranged widely from 0.096 to 4.32 mg N-NO_2_^−^ kg^−1^ 24 h^−1^ (Table 11). The highest activity was observed in 2011 (mean 2.55 mg N-NO_2_^−^ kg^−1^ 24 h^−1^) followed by 2009 and 2010 (mean 0.39 mg N-NO_2_^−^ kg^−1^  24 h^−1^) (Fig. 2d). There was no consistent trend in the NR activity in individual years as affected by sampling dates (Table 11). The only one clear trend was that the NR activity was the highest in May during the entire experimental period. As compared with the control, the application of the catch crop significantly influenced the NR activity in 2009 (except in June) and in 2011 (except in August). The time of the catch crop incorporation markedly affected the NR activity only in March 2009, while at other sampling times the activity was not influenced or no clear trends were observed in this activity when the individual years of the study were examined.

**Table 11:** Nitrate reductase activity (NR) (mg N-NO_2_^−^ kg^−1^ 24 h^−1^) as dependent on the time of catch crop incorporation and sampling season

Catch crop	^§^March	May	June	August
2009				
A^&^	0.158 b^C^#^	0.920aA	0.721 abA	0.307aB
B	0.180 aC	0.850aA	0.888 aA	0.331aB
C	0.096 cC	0.705bA	0.605 bA	0.255bB
Mean	0,145C	0.825A	0.738A	0.298B
2010				
A	0.286abA	0.330 abA	0.266 abA	0.266aA
B	0.355aA	0.379 aA	0.298 aA	0.242aA
C	0.207bA	0.268 bA	0.223 bA	0.127bB
Mean	0.283A	0.326A	0.262A	0.212A
2011				
A	2.90 aB	4.32aA	1.86 abC	1.86aC
B	2.80 aB	4.07aA	2.08 aB	1.63aC
C	2.39 bB	3.46bA	1.55 bC	1.61aC
Mean	2.70B	3.95A	1.83C	1.70C
2009–2011				
A	1.30 aA	1.86 aA	0.95 bB	0.81aB
B	1.35 aAB	1.77 bA	1.09 aB	0.73aC
C	1.07 bAB	1.48 cA	0.79 cB	0.66aB
Mean	1.24B	1.70A	0.94B	0.74B

^&^A - catch crop incorporated in autumn, B – catch crop incorporated in spring, C – control (without a catch crop); ^§^ sampling dates

Values followed by the same small letter within each column are not significantly different at *P* <  0.05. Values followed by the same capital letter within a line are not significantly different at *P* <  0.05.

^ Different small letters indicate comparison between catch crops treatments (within the same sampling date).

^#^ Different capital letters indicate a comparison among sampling dates within the same catch crop treatments.

### Relationship between the properties studied

According to multiple linear regression analysis, the soil enzymes were significantly correlated with some chemical properties (Table 12). A significant correlation was found between the total and nitrate-N contents, C_ORG_ content, pH_KCl_ and the N-cycle enzymes. The enzyme activities were positively correlated with pH_KCl_ and the MBN negatively. The N-NO_3_^−^ content was significantly and positively related to PRO and NR and negatively with AAR. The activity of UR and AAR was negatively related to the ammonium-N content. The highest correlation coefficients (r^2^ = 0.682 - 0.811) were observed between NR activity and N_TOT_ and C_ORG_ content at every variant of catch crop management (Table 13). A closer relationship between NR activity and N-NO_3_^−^ content was found when the catch crop was incorporated in the autumn (r^2^ = 0.596) followed by the spring incorporation and the control (r^2^ = 0.454 and 0.219, respectively).

## Discussion

### Basic chemical properties

Generally, neither catch crop management and the time of its incorporation as well as sampling season had an influence on the organic carbon and total nitrogen content or soil reaction during the three-year-long experimental period (Tables 4 and 7). In this study the lack of the temporal accumulation of C_ORG_ and N_TOT_ took place because the experiment was carried out in a different section of the field each year and one year of the study might be too short to detect differences in C_ORG_ and N_TOT_ due to catch crop treatment. The results related to the effect of catch crops on the C_ORG_ and N_TOT_ content in different experiments were contradictory; some results suggest an increase in these properties, but often no changes were found. In the study of Navas et al. (2011), total nitrogen was among the chemical variables that were influenced the most by the legume cover crops that were evaluated. Prior to the experiment, the average concentration of the N_TOT_ in soil was 0.1 g kg^−1^, while after three years of soil cover with legumes, this value increased to 0.4-0.8 g kg^−1^. In another study (Berntsen et al. 2006), the content of N_ORG_ was only 10 % higher when the catch crop was applied compared to the control as well as when it was incorporated in the spring rather than in the autumn. In contrast, other data revealed that cover crops did not have any significant effect on changes in the soil N_TOT_ content two years after their application as green manure (Arlauskiené and Maikšténiené 2010). The lack of differences in the soil organic carbon content as influenced by different catch crops compared to the control was also observed during a period of three years (Eichler-Löbermann et al. 2008) or after eight years of a high input of organic matter in the form of pig slurry and catch crops (Debosz et al. 1999). This indicates a rapid degradation of cover crop residues when incorporated into the soil as stated by Kuo et al. (1997). According to the authors, the rapid decay of the cover crops after incorporation into the soil and the six years of continuous winter cover cropping had only a limited effect on the soil C_ORG_ content.

### Soil mineral nitrogen

A higher content of the mineral N (especially N-NO_3_^−^) after the spring incorporation compared with the autumn application may be due to the partial loss of the mineral N during the autumn and winter. When the catch crop was incorporated in the autumn, partial mineralization might have taken place because of temperatures above zero (4-5 °C) and the relatively high precipitation in November (2008–2010) (Table 2). A general assumption has been that N mineralization is low when the soil temperature is below 5 °C (Thorup-Kristensen et al. 2003). Additionally, in this study the mineralization processes in the autumn/winter period might have been enhanced due to the sandy-loam soil, which had good air conditions. When the catch crops were left for winter, the mineralization was limited firstly by the growth of the catch crop and secondly because of the low temperatures that occurred from December to February that damaged the green biomass (Table 2). The major mineralization of the incorporated biomass happened in the spring, which was confirmed by the fact that the highest enzymatic activity occurred in May and June (Tables 8, 9, 10 and 11, Fig. 4).

As was shown in earlier studies (Känkänen et al. 1998), the N content of the incorporated catch crop had a marked effect on the mineral N content after incorporation. In this study the highest biomass of the catch crop that was observed in 2008 (Fig. 1) was clearly related to the highest N uptake in that year (Table 3) and the highest yield of spring barley in 2009 (data not presented), but not with the mineral N content in subsequent years. The only consistent trends were that the highest concentration (% of dry matter) in 2010 was related to the highest content of N-NO_3_^−^ in the soil in 2011. Moreover, the highest N uptake (Table 3) and catch crop yield in 2008 (Fig. 1) corresponded to the lowest N-NO_3_^−^ content in 2009. In this case nitrate ions could have been partially leached and/or accumulated in the spring barley yield.

In this study there was no clear seasonal pattern for the mineral nitrogen content. Usually the nutrient content in soil that is available to plants after the main crops are harvested is usually lower than in the spring before crop cultivation (Eichler-Löbermann et al. 2008), which was true in this study for N-NO_3_^−^ in 2010 and 2011. During the growth of legume catch crops, the nutrient content in the soil is usually lower due to the biological nutrient fixation in the plant biomass. After incorporation into the soil, the nutrients in the plant biomass are released during the process of decomposition, which is most intensive in the spring and at the beginning of summer, i.e., in the period of the most intensive spring crop growth. The decrease in mineral N in August may have been due to the fact that part of mineral N was accumulated in the yield of sequent crop and the biological processes of mineralization in soil decreased because the growth of plants was finished. In this study the N effect of the catch crop could have been masked by the fact that the mineral N fertilization was applied every year before the sowing and during the shooting of spring barley. High ammonium-N in June in 2009 and 2010, compared to other sampling months, could have been due to the N-fertilization that had been conducted almost one month earlier.

The content of the soil mineral nitrate N in the spring (March) may have been influenced by the mineralization of organic matter and the leaching of N during the late autumn, winter and early spring. The rate of mineralization is governed not only by the type of organic matter in the soil, but also by the soil temperature, moisture content, soil acidity, soil aeration and other factors (Camberato 2001). Crozier et al. (1998) found 45 % of cover crops N mineralized shortly after the incorporation of green manure crops. In the following months (December-February), the mineralization may be decreased because low temperatures (near or below 0 °C) slow down the decomposition of plant materials and the nitrification in soil, thus suggesting low mineralization and leaching during the winter when the soil is usually frozen (Känkänen et al. 1998). On the other hand, mineralization at low temperatures is not negligible and a high release of N from the incorporated plant material was obtained during the period when the soil was frozen most of the time (Müller and Sundman 1988). The occurrence of the leaching process in the autumn/winter period (mainly in November) in this study could have been indirectly confirmed by the fact that the content of N-NO_3_^−^ in October (before the catch crop incorporation) was almost 20-30 % higher than in March in the subsequent years (data not shown). In March 2009 the precipitation was higher than in March 2010 and 2011, which was clearly connected with the content of N-NO_3_^−^ in the soil at the end of March in subsequent years. Due to the higher precipitation in March, a lower content of N-NO_3_^−^ in the soil was found in this month. Similarly, a high precipitation level in August 2010 and in June and July 2011 caused a lower soil N-NO_3_^−^ content, which could have been connected with partial leaching. The adequate explanation was given by Panek (1993), who stated that in the Midwestern Poland (area of soil sampling ) spring barley in June and July (earing and wax maturation) is able to uptake no more than 80 mm of water head per month. In June and July 2011 precipitation was higher that this value (Table 2) and therefore we can presume that partial leaching took place. The other reason of low soil N-NO_3_^−^ content in May 2009 and June 2010 and 2011was probably its accumulation in the yield of spring barley dependent on its well developed root system. Indeed, the lowest content of N-NO_3_^−^ in the soil was found in 2009 for which the highest yield of spring barley was obtained (data not presented). The increased content of N-NO_3_^−^ in May that was found in the present experiment (2010 and 2011) was probably caused by the mineralization of plant residues in the early spring or could be partially caused by the first dose of N-fertilizer applied.

Ammonium-N can be lost from the field during the volatilization of ammonia gas (NH_3_) or can be converted to nitrate by soil bacteria. The rate of the nitrification and volatilization processes is affected by soil moisture and temperature. Additionally, the bacteria involved in the nitrification are very sensitive to soil pH and the higher the soil pH, the higher the nitrification rate (Gieseke et al. 2006). The optimum pH for this process may vary from 6.6 to 8.0 (Tarre et al. 2004). In this study there was only optimum pH for the nitrification (6.75) in 2011 (Table 7) and, in fact, the highest content of N-NO_3_^−^ (13.2 mg kg^−1^) together with the lowest N-NH_4_^+^ concentration (2.94 mg kg^−1^) was observed in that year (Tables 5 and 6). In addition, the values of pH_KCl_ were significantly and positively correlated with the N-NO_3_^−^ content and negatively with the N-NH_4_^+^ content (data not presented) which confirms the statement above.

### Microbial biomass nitrogen and the activity of N-related enzymes

The microbial biomass and activity are the main biological indicators of soil quality and respond rapidly to changes resulting from agronomic practices (Araújo et al. 2003). Soil enzymes are the primary mediators of soil biological processes, including organic matter degradation, mineralization and nutrient cycling (Li et al. 2009). Higher PRO and NR activity and MBN content in catch crop treatment as compared to the control probably resulted from the input of the catch crop biomass. Soil microorganisms degrade organic matter through the production of different extracellular enzymes and for this reason after the application of green manures to soil, the enzymatic activity of the soil increases (Tejada et al. 2008). The fresh plant biomass provides readily accessible substrates for microorganisms and the specific quality of the organic residues controls the decomposition rate and the release of nutrients by soil microorganisms (Arlauskiené and Maikšténiené 2010). Leguminous catch crops, because of their low C:N ratio, especially contain easily available compounds, such as amino acids and carbohydrates, which can stimulate the microbial population and its activity (Kumar and Goh 2003; Dinesh et al. 2004). Moreover, the added green biomass may not only stimulate microbial activity but also may contain intra- and extracellular enzymes as stated Pascual et al. (1998). The lower or unaffected activity of UR and AAR when the catch crop was applied as compared to the control was probably caused by fact that the activity of the soil enzymes involved in the cycling of a given nutrient are often negatively related to the availability of that nutrient in the soil (Dick 1992). In our study the activity of UR and AAR was negatively correlated with the N-NH_4_^+^ content (Table 12), which can be explained by the fact that the synthesis of these enzymes is repressed when the cells are grown in the presence of a preferred N source such as N-NH_4_^+^ (Marcote et al. 2001; Geisseler et al. 2010).

The significantly higher biomass N content when the catch crop was incorporated in the spring as compared to autumn incorporation (Fig. 2a) was probably caused by the fact that the spring incorporation allowed more time for the catch crop to grow. The amount of the catch crop biomass incorporated in the spring was probably higher than that applied in the autumn. The incorporation of a higher catch crop biomass in the spring initiated an intensive decomposition process, due to the higher temperatures that occurred in the spring months and at the beginning of spring barley growth, which could have promoted the growth of indigenous microorganisms. The catch crop incorporated in the autumn was already at a more advanced mineralization stage and in the spring the intensity of the decomposition would have been lower. Since the enzymatic activity was either unaffected by the time of the catch crop incorporation or because the results were inconsistent, other factors may have influenced the activity. The activity might have been the result of opposite effects that were dependent on the relative amounts of the substrates and products that were presents, which could have regulated the activity.

Seasonal changes in soil enzymes could have been related to the intensity of the catch crop mineralization as well as to the temperature and soil moisture. In fact, a marked interaction between the temperature data and enzymatic activity on subsequent sampling dates was found in the study. The assayed enzymes showed a higher activity in May and June (except for NR activity) than in March when the temperature was clearly lower (Table 2). However, there was not such a relationship between the enzymatic activity and temperature data in August. Similarly, no marked relationship was found between enzyme activities and the level of precipitation in the periods of the sampling. The period of 1^st ^to 10^th^ August 2009 was very dry while during the same period in 2010 it was rather wet (81 mm) (detailed data of precipitation are not presented). However, this was not reflected in any enzyme activity. It is probable that the enzymatic activity patterns were more affected by the catch crops mineralization that was discussed above or other agents rather than by climatic factors.

Although the content of C_ORG_ and N_TOT_ was not significantly influenced by catch crop management and the sampling season (except C_ORG_ in 2009), the enzymatic activity was significantly correlated with these properties (Tables 12, 13), which have been described earlier in other soils (e.g. Chaer et al. 2009; Chodak et al. 2003; Nsabimana et al. 2004). In fact, the C_ORG_ and N_TOT_ content plays an important role in determining the size of the microbial biomass and the level of enzyme activity (Chaer et al. 2009). A significant relation between NR activity and N-NO_3_^−^ (Tables 12, 13) was expected since nitrate reductase is an adaptive enzyme and is only synthesized in the presence of NO_3_^−^ ions, while it is repressed by NH_4_^+^ ions in a soil solution (Rice and Tiedje 1989; McCarty and Bremner 1992).

**Table 12:** Regression summary for dependent variables (y)

Dependent variables (y)	Regression	*p*	r^2^
UR	y = 5.23 + 1.175 C_ORG_ – 0.456 N-NH_4_^+^ ± 2.03	0.00130	0.223
PRO	y = 38.9 + 6.2 N_TOT_ + 5.03 C_ORG_ + 5.05 pH_KCl_ + 0.8 N-NO_3_^−^ ± 6.69	0.00052	0.314
AAR	y = −1.43 + 3.08 N_TOT_ + 1.04 C_ORG_ +0.469 pH_KCl_ – 0.033 N-NO_3_^−^ – 0.130 N-NH_4_^+^ ± 0.451	0.04940	0.376
NR	y = −2.27 + 0.156N_TOT_ + 0.385 C_ORG_ + 0.453 pH_KCl_ + 0.048 N-NO_3_^−^ ± 0.384	0.00042	0.854
MB-N	y = 34.1 + 38.0 N_TOT_ + 2.76 C_ORG_ – 3.99 pH_KCl_ + 0.321 N-NO_3_^−^ ± 5.03	0.00603	0.177

**Table 13:** Regression summary for dependent variable (y) NR

Variables	Catch crop management	Regression	*p*	r^2^
NR x N_TOT_	A	y = −2.06 + 4.01x ± 0.608	0.000000	0.702
	B	y = −2.14 + 4.10x ± 0.568	0.000000	0.682
	C	y = −1.92 + 3.68x ± 0.471	0.000321	0.743
NR x C_ORG_	A	y = −2.81 + 0.423x ± 0.554	0.000000	0.752
	B	y = −3.29 + 0.447x ± 0.496	0.000000	0.757
	C	y = −2.56 + 0.379x ± 0.403	0.000000	0.811
NR x N-NO_3_^−^	A	y = 0.352 + 0.156x ± 0.545	0.000256	0.596
	B	y = 0.195 + 0.095x ± 0.645	0.000332	0.454
	C	y = 0.169 + 0.088x ± 0.891	0.000956	0.219

## Conclusions

Our study showed that use of field pea as a green manure, in contrast to the control soil, significantly increased the soil mineral and microbial biomass N content as well as the activities of protease and nitrate reductase during the growing period of the following crop. The spring incorporation of the catch crop significantly increased the MBN content in contrast to the autumn application, while the activity of N-cycle enzymes was clearly unaffected by the time of the incorporation of field pea or the results were inconsistent. When field pea was incorporated in the spring, a significantly higher content of mineral N, as compared to the autumn incorporation, was only observed on two of the four sampling dates (mean for 2009–2011). The higher content of mineral N in treatments with the catch crop than in the control indicated the significant potential of a catch crop in reducing the leaching of nitrate-N from the topsoil. When the catch crop was incorporated in autumn, the partial mineralization of organic matter and leaching of N-NO_3_^−^ during the autumn/winter period might have occurred.

In conclusion, the results obtained indicate that the application of a catch crop can be recommended as a means of increasing the soil biological activity and the content of mineral N in the soil. Since there was an ambiguous effect related to the time of the catch crop incorporation for most of the properties studied, i.e., there was no significant effect or no consistent results were obtained, both a spring and autumn application can be recommended as a management tool to improve the status of soil properties during the growth of the sequent crop. It is, however, important to highlight the fact that soils from different origins and properties may respond differently to the time when the catch crop is incorporated.
